# Immunophenotypic Detection of Measurable Residual (Stem Cell) Disease Using LAIP Approach in Acute Myeloid Leukemia

**DOI:** 10.1002/cpcy.66

**Published:** 2019-10-23

**Authors:** Wendelien Zeijlemaker, Angele Kelder, Jacqueline Cloos, Gerrit Jan Schuurhuis

**Affiliations:** ^1^ Department of Hematology Amsterdam University Medical Center, Cancer Center VU University Medical Center Amsterdam The Netherlands

**Keywords:** acute myeloid leukemia (AML), leukemia associated immunophenotypes (LAIPs), leukemic stem cells (LSCs), measurable residual disease (MRD), multiparameter flow cytometry (MFC)

## Abstract

Half of the patients with acute myeloid leukemia (AML), who achieve complete remission after chemotherapy treatment, will ultimately experience a relapse. Measurable residual disease (MRD) is an important post‐treatment risk factor in AML, because it gives additional information about the depth of the remission. Within MRD, the small population of leukemic stem cells (LSCs) is thought to be at the base of the actual relapse. In this protocol, the flow cytometric detection of MRD and LSCs herein is outlined. We give a detailed overview of the sampling procedures for optimal multiparameter flow cytometry assessment of both MRD and LSC, using leukemia associated immunophenotypes (LAIPs) and LSC markers. Moreover, an overview of the gating strategies to detect LAIPs and LSC markers is provided. This protocol serves as guidance for flow cytometric detection of measurable residual (stem cell) disease necessary for proper therapeutic decision making in AML patients. © 2019 The Authors.

**Basic Protocol 1**: Immunophenotypic LAIP detection for measurable residual disease monitoring

**Basic Protocol 2**: Immunophenotypic detection of CD34+CD38− leukemic stem cells

## INTRODUCTION

Acute myeloid leukemia (AML) is a malignancy of the bone marrow (BM) characterized by abnormal accumulation of immature progenitor cells and inhibition of normal hematopoiesis. Treatment strategies consist mainly of chemotherapy treatment and autologous or allogeneic stem cell transplantation. Despite current risk‐adapted treatment strategies, a considerable number of AML patients will still experience relapse. Further improvement of risk definition in AML is of utmost importance because this would enable better risk‐adapted treatment strategies with improved patient outcome. Measurable residual disease (MRD), defined as post‐therapy persistence of leukemic cells, is currently one of the well‐established risk factors in AML. This article is primarily aimed at detection of MRD in AML patients. The focus will be on identification of so‐called leukemia associated immunophenotypes (LAIPs) defined at diagnosis and applied in follow‐up. In multiple studies, MRD was found to be an independent prognostic factor that gives important additional information about the depth of the remission after treatment (Buccisano, Hourigan, & Walter, 2017). In Basic Protocol 1, the approach of immunophenotypic MRD detection is described. Within MRD, the small population of therapy resistant leukemic stem cells (LSCs) is suggested to be at the base of the actual outgrowth of residual cells to relapse and the frequency of these LSCs is also of prognostic significance (Zeijlemaker et al., 2019). Although these stem cells can have different immunophenotypes, the focus here is on the CD34+CD38− LSC. In Basic Protocol 2, the approach of detection of CD34+CD38− LSCs, as part of the total MRD population, is described. These protocols are designed to enable accurate and reproducible immunophenotypic detection of measurable residual (stem cell) disease in AML.

## STRATEGIC PLANNING

### Transport/Storage Conditions

(1) When AML samples arrive from other institutes, correct packaging at the local institute must be ensured. To enable this, a “gel‐pack” can be used to prevent the tubes from breaking and leaking. Moreover, the bone marrow (BM) samples are best kept at room temperature during transport to ensure optimal cell viability. For more details concerning transport of material, see the recent video article by Cloos et al. (2018).

(2) Bone marrow or peripheral blood samples that are not processed the same day can be kept overnight at room temperature. The tubes are preferably stored in a horizontal position.

(3) For diagnosis material, storage of the AML sample up to 3 days (and in most cases up to 5 days) is allowed. For follow‐up material, it is recommended that the assays are processed and performed the same day, because the influence of storage time on the actual residual disease percentage is largely unknown.

### Choice of Antibody Combinations

The selection of antibodies is of major importance for proper residual disease detection. Therefore, research efforts have focused on cell surface markers to identify different leukemic (sub)clones at the time of diagnosis (Boer de et al., 2018). However, many different markers have been used in different AML studies for residual (stem cell) disease identification. In a recent consensus article by the European LeukemiaNet MRD Working Party, guidelines are given for appropriate composition of MRD panels (Schuurhuis et al., 2018). This document aims for consensus for a range of parameters on how to harmonize MRD detection in AML. The ultimate goal is one standardized MRD panel for all MRD studies in AML (Schuurhuis et al., 2018). Table 1 (used in Basic Protocol 1) shows the MRD antibody panel as used in several studies of the clinical Hemato Oncology Foundation for Adults in the Netherlands (HOVON)/Swiss Group for Clinical Cancer Research (SAKK) consortium and in industrial studies that are performed predominantly in Amsterdam. Table 2 (used in Basic Protocol 2) shows the currently used stem cell tube in these protocols. For details concerning the composition of this one‐tube LSC assay, refer to our corresponding paper (Zeijlemaker et al., 2016b).

**Table 1:** Measurable Residual Disease (MRD) Antibody Panel

Tube	FITC	PE	PerCP‐CY5.5	PC7	APC	APC‐H7	BV421	KO
1	CD7	CD56	CD34	CD117	CD33	HLA‐DR	CD13	CD45
2	CD15	CD22	CD34	CD117	CD19	HLADR	CD13	CD45
3	CD36	CD14	CD34	CD117	CD11b	HLADR	CD13	CD45
4	CD2	CD133	CD34	CD117	CD33	HLADR	CD13	CD45

**Table 2:** Leukemic Stem Cell (LSC) Antibody Panel

Tube	FITC	PE	PerCP‐CY5.5	PC7	APC	APC‐H7	BV421	KO
1	CD45RA	Clec12a	CD123	CD34	CD38	CD44	CD33	CD45
		TIM‐3						
		CD7						
		CD11b						
		CD22						
		CD56						

## Basic Protocol 1 IMMUNOPHENOTYPIC LAIP DETECTION FOR MEASURABLE RESIDUAL DISEASE MONITORING

This protocol describes immunophenotypic MRD detection using the leukemia associated immunophenotypes (LAIPs) approach. Appropriate instrument settings are crucial for performing adequate flow cytometric assays; this topic is beyond the scope of this protocol. A flow cytometer setup guideline, including instructions concerning compensation settings, can be found in the video article by Cloos and colleagues (2018). Details are also provided by the EuroFlow guideline for standardization of instrument settings (Kalina et al., 2012).

### Materials

10 ml bone marrow in heparin (e.g., lithium heparin, 102 IU coated tubes; Becton Dickinson) or EDTA tube (e.g., plastic K2EDTA 7.2 mg; Becton Dickinson)
Türk cell staining solution (MilliporeSigma, cat. no. 1092770100)
PBS (see recipe)
Measurable residual disease (MRD) antibody panel (see Table 1)
Lysing solution (see recipe)

Cell counting chamber or automated cell counter (e.g., Cellometer Spectrum, Nexcelom)
Tabletop centrifuge
Multicolor flow cytometer
Software (e.g., Infinicyt, Flowjo)
4 FACS tubes
Pipets
Filter pipet tips

### Prepare bone marrow sample

1 Assess concentration of white blood cells (WBCs) in the BM sample using a cell counting chamber with Türk cell staining solution by dissolving 10 µl BM in 90 µl Türk solution. Mix gently and incubate ∼1 min. Fill cell counting chamber and define cell concentration.
  Alternatively, an automated cell counter (e.g., Cellometer Spectrum, Nexcelom) can be used to determine the cell concentration and viability.
2 Define volume of BM needed and pipet this volume into a separate regular 15‐ml tube. For one tube (see Table 1) use ∼500,000 white blood cells (for the four tubes, 2 × 10^6^ cells). For follow‐up samples, use 2 × 10^6^ cells per tube (for the four tubes, 8 × 10^6^ in total).
3 Add lysing solution to the tube containing white blood cell suspension to lyse any red blood cells.
  The volume of lysing solution should be ten times the volume of the white blood cell suspension.
  Preferably for bulk lysing, use one tube (e.g., 50 ml).
4 Mix gently by inverting the tubes and incubate 10 min at room temperature then centrifuge 7 min at 700 × *g* (room temperature with mild brake, e.g., Hettich Rotolavit centrifuge brake 3).
5 Remove supernatant and re‐suspend cell pellet in excess PBS. Centrifuge 7 min at 700 × *g* (room temperature with mild brake).
6 Remove supernatant. Re‐suspend cell pellet in PBS to a cell concentration of ∼100 × 10^6^ WBC/ml before dividing cell suspension evenly over the four different FACS tubes.

### Stain white blood cells

7 Pipet appropriate monoclonal antibodies into the different tubes (detailed in Table 1; eight different antibodies per tube). Mix gently and incubate cell suspensions (20 µl) with the appropriate antibodies (20 µl premix containing all eight antibodies) 15 min at room temperature while protecting from light.
  Each antibody must have been titrated on appropriate control cells by the laboratory itself, to assess the optimal concentrations of the antibodies.
8 Add 3 ml PBS per tube to wash the stained cells. Centrifuge cells at 400 × *g* for 5 min (with brake). Remove supernatant and re‐suspend cell pellet in 300 µl PBS.

### Flow cytometry LAIP assessment at diagnosis

9 Use the flow cytometer to measure at least 100,000 gated WBCs per tube for diagnosis samples.
  Typically, due to losses in the procedures, 200,000‐300,000 cells are available. LAIP assessment at diagnosis is required for proper identification of residual LAIP positive cells at follow‐up.
  Measure all four tubes to enable full LAIP identification.
10 Save FACS data using an appropriate file name (e.g., MRD‐diagnosis‐BM‐patient number 1‐date sample measurement‐LAIP CD34/CD13/CD56).

### Flow cytometry LAIP assessment at follow‐up

11 Use the flow cytometer to measure at least 1,000,000 gated WBCs for follow‐up samples.
12 Usually there is enough BM at follow‐up to use all four regular tubes for complete LAIP follow‐up identification and to enable detection of upcoming LAIPs. Exceptions are possible; these include absence of LAIPs at diagnosis or absence of diagnosis information:
  - In case of limited amount of BM cells, use tube(s) that allow determination of LAIP(s) that were present at diagnosis of AML.
  - In rare cases both with no diagnosis of LAIP available and with shortage of follow‐up material, only one, two, or three tubes can be measured at follow‐up. Based on the frequencies of LAIPs, as used in the recent HO102 study (Zeijlemaker et al., 2019), the chances of finding a good LAIP (tube numbers shown in Table 1) are 59% for tube 1, 20% for tube 2, 11% for tube 3, and 10% for tube 4. So, with such a shortage of material and with no diagnosis information, the preferential choice would be tube 1, followed by tube 2, followed by tube 3 and tube 4.
  - Importantly, if there is evidence for a CD34‐negative AML (in most cases defined already at diagnosis), at least tube 4, with the antibody for the primitive marker CD133 present, should be used.
  - Note that some upcoming LAIPs (defined as LAIPs present at follow‐up that were not, or in very low frequency, present at time of diagnosis), representing upcoming leukemic populations, can be missed when not all four tubes are measured at follow‐up.
13 Save FACS data using an appropriate file name (e.g., MRD‐follow‐up‐BM‐patient number 1‐date sample measurement‐LAIP CD34/CD13/CD56).

### Gating strategy to identify LAIPs at diagnosis and follow‐up

14 Open FACS data from the diagnosis files using gating software to assess diagnosis of LAIPs. Use these diagnosis files at follow‐up to assess both the LAIPs defined at diagnosis to be important for follow‐up, as well as LAIPs that were not present at diagnosis but emerged during and/or after treatment.

#### For white blood cells

**Figure 1:**
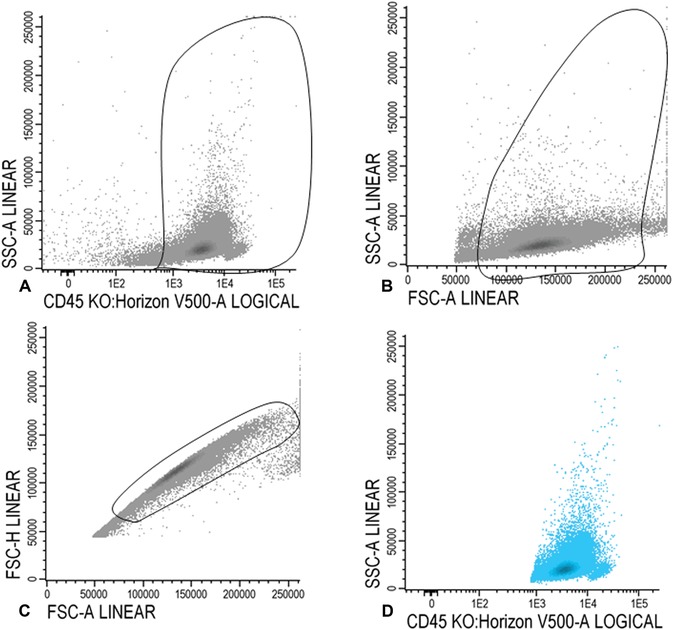
Gating white blood cells. (**A‐C**) Gating of the white blood cells. For a detailed description of the gating steps see Basic Protocol 1, step 15a. (**D**)The final population of white blood cells is shown in blue in D.

15a Gate CD45 positive cells in the CD45/SSC plot (Fig. 1A). In the FSC/SSC plot, set a gate on the larger cells, thereby gating out the red cells, debris, and cells with very high FSC (Fig. 1B). Figure 1C illustrates gating of the single cells and removal of doublets in an FSCA/FSC‐H plot. All WBCs are shown in Figure 1D.

#### For lymphocytes

**Figure 2:**
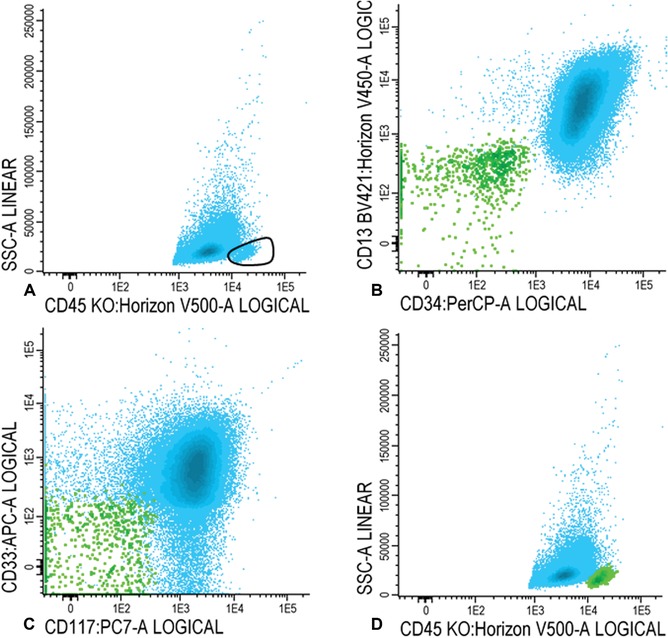
Gating lymphocytes. (**A**) Gating of the lymphocytes. (**B‐D**) Lymphocytes are shown in green in B‐D. See Basic Protocol 1, step 15b for a detailed description of the gating steps.

15b Within the population of WBCs (blue), as shown in Figure 1D, gate CD45 high and SSC low cells (Fig. 2A). Ensure that there are no myeloid cells in the gate using CD34, CD117, CD13, and/or CD33.
  Examples of the absence of expression of these four markers is shown in Figure 2B and C. The final population of lymphocytes is shown in green in Figures 2B‐D. Lymphocytes can be used as an internal control to define marker expression (e.g., use the CD7‐negative population of lymphocytes to define CD7‐positivity on leukemic blasts).

#### For immature blasts

**Figure 3:**
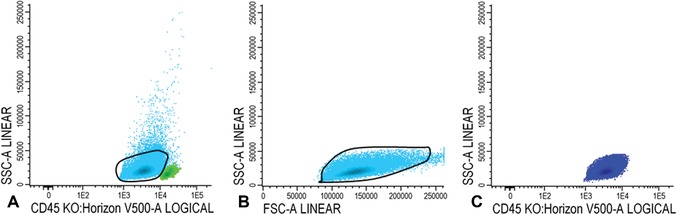
Gating immature blasts. (**A‐B**) Gating of the immature blast cells. See Basic Protocol 1, step 15c for a detailed description of these gating steps. (**C**) The final population of immature blast cells is shown in dark blue.

15c Within the WBC fraction (shown in blue in Fig. 2D), gate immature blasts in the CD45/SSC plot (Fig. 3A). Blasts are CD45 intermediate and have a low SSC.
  Lymphocytes are shown in green (Fig. 3A).
  Use the FSC/SSC plot to ensure that mature granulocytes or immature lymphocytes have been excluded from the immature blast fraction (Fig. 3B); the final population of immature blasts cells is shown in dark blue in a CD45/SSC plot in Figure 3C.

#### For LAIP positive cells at diagnosis

**Figure 4:**
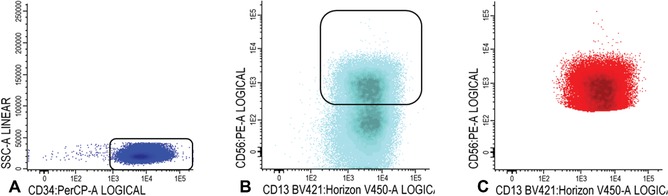
LAIP detection on immature blasts at diagnosis. (**A‐B**) Gating of LAIP positive cells. For a detailed description of the gating steps see Basic Protocol 1, step 16. (**C**) LAIP positive immature blasts are shown in red. LAIP, leukemia associated immunophenotype.

**Figure 5:**
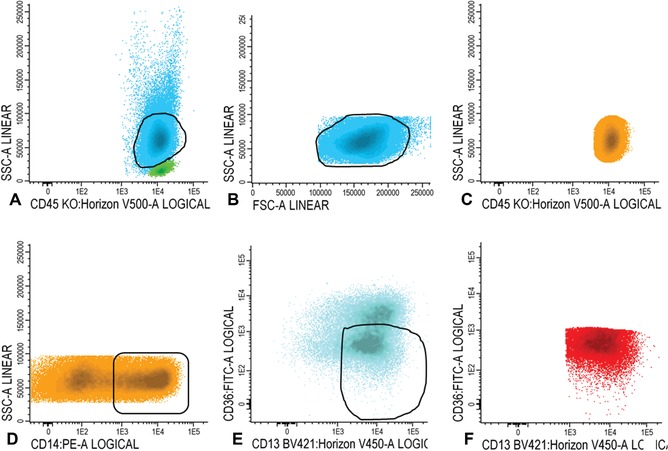
LAIP detection on mature blasts at diagnosis. (**A‐B**) Gating of mature blast cells. (**C**) The population of mature blast cells is shown in orange. (**D**‐**E**) Gating of LAIP positive cells. See Basic Protocol 1, step 16 for a detailed description of these gating steps. (**F**) LAIP positive mature blasts are shown in red. LAIP, leukemia associated immunophenotype.

16 Within the immature blast fraction (Fig. 3C), identify primitive marker positive blasts cells (CD34, CD117, or CD133 positive cells; example with CD34 shown in Fig. 4A). Plot primitive marker positive cells (in Fig. 4B, light blue represents all CD34 positive blast cells) in a plot with a positive myeloid marker (CD13, CD33, or HLA‐DR positive cells; example with CD13 shown in Fig. 4B) and search for an LAIP.
  An example with CD56 is shown in Figure 4B and this shows that part of the CD34+CD13+ cells represents the LAIP CD34+CD13+CD56+ and is shown in red in Figure 4C. In this example the LAIP covers 47.1% of the total amount of immature blast cells.
  In a small fraction of AML patients, the leukemic blasts do not express a primitive marker (CD34, CD117, or CD133). In these cases, more mature LAIPs may be detectable. Figure 5 shows an example of such a “mature” LAIP. Figure 5A and B show gating of the mature blast cells. In Figure 5C, the mature blasts are shown in orange. Figure 5D shows that part of the mature blast cells are CD14 positive. Subsequently, Figure 5E shows all CD14 positive mature blast cells in light blue whereby staining for CD36 reveals that part of the CD13 positive cells are negative for CD36. Finally, the CD45+/CD14+/CD13+/CD36− LAIP positive cells are shown in red in Figure 5F. In this example, the LAIP covers 25.2% of the total amount of mature blast cells.

#### For LAIP positive cells at follow‐up

**Figure 6:**
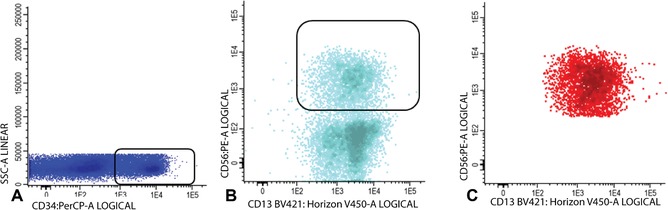
LAIP detection at follow‐up. (**A‐B**) Gating of LAIP positive cells in follow‐up BM. See Basic Protocol 1, step 17 for a detailed description of these gating steps. (**C**) LAIP positive blasts are shown in red. LAIP, leukemia associated immunophenotype; BM, bone marrow.

17 Gate lymphocytes and (im)mature blasts as described in steps 14 and 15a‐15c. Similar to step 16, check for the presence of all possible LAIPs. First, focus on the LAIP phenotype that was established at diagnosis. Choose the best LAIP based on the percentage of LAIP positivity and on LAIP specificity (see also the Commentary section; in this example we use CD45/CD34/CD13/CD56 LAIP).
  In Figure 6A‐C, the same gating steps as described in step 16 for diagnosis (Fig. 4) are illustrated for follow‐up BM. In this case, we defined the sample as MRD positive, because LAIP positive cells were 1.80% of the total amount of WBCs which is above the presently defined European LeukemiaNet (ELN) consensus cut‐off of 0.1% (Schuurhuis et al., 2018); thus, this AML BM sample was defined as MRD positive. A similar approach is used for mature LAIPs, although detection of mature LAIPs in follow‐up BM is challenging and requires some gating experience.

## Basic Protocol 2 IMMUNOPHENOTYPIC DETECTION OF CD34+CD38− LEUKEMIC STEM CELLS

An overview is given of the approach of immunophenotypic detection of CD34+CD38− leukemic stem cells. We developed an antibody panel that allows the assessment of the total CD34+CD38− LSC load using only one tube (Zeijlemaker et al., 2016b). The content of this tube is shown in Table 2. It is characterized by a cocktail of six different antibodies in the phycoerythrin (PE) channel (referred to as Combi‐6 channel) that, together with CD45RA, CD123, CD44, and CD33, allows the assessment of total LSC load efficiently and accurately in the majority of AML patients. More information concerning the limitations of this approach is given in the Commentary section.

### Additional Materials (see also Basic Protocol 1)

Leukemic stem cell (LSC) monoclonal antibodies (see Table 2)

### Prepare bone marrow sample

1 Prepare bone marrow cells for flow cytometry measurement as described in step 1 of Basic Protocol 1.
  Use 8 × 10^6^ WBCs (or more if possible) for measurement of stem cells.

### Staining of white blood cells

2 See step 2 of Basic Protocol 1.
  Use 500 µl to re‐suspend the cell pellet to prevent excessively high flow rates in the flow cytometer due to the higher cell number used for stem cell analyses.

### Flow cytometry LSC assessment

3 Use a flow cytometer to measure as many gated WBCs as possible, at least 4 × 10^6^, especially for follow‐up samples.
  High WBC counts are recommended to enable proper LSC detection.
4 Save FACS data using an appropriate file name, e.g., LSC‐diagnosis‐BM‐patient number 1‐date sample measurement‐Marker Combi‐6.

### Gating strategy to identify CD34+CD38−LSCs at diagnosis and follow‐up

**Figure 7:**
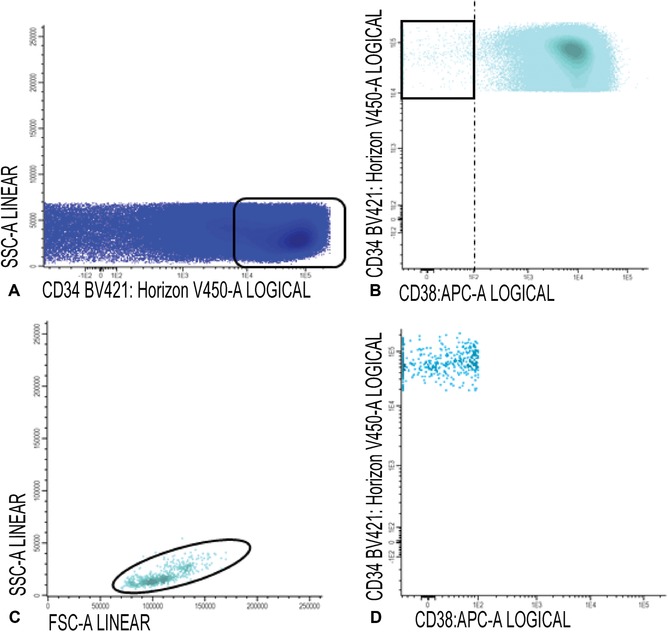
Gating of CD34+CD38− cells. (**A‐C**) Gating of the CD34+CD38− cells. For a detailed description of the gating steps see Basic Protocol 2, step 6. (**D**) The population of CD34+CD38− stem cells is shown in azure blue.

5 Gate white blood cells, lymphocytes, and immature blast cells as described in steps 14‐15 of Basic Protocol 1.
6 CD34+CD38− cells: Gate CD34 positive blast cells as shown in Figure 7A; CD34 positive blasts are shown in light blue in Figure 7B, where CD34 is plotted against CD38. Identify CD38 low cells (also referred as CD38− cells; gate in Fig. 7B). To facilitate the detection of CD38 low cells, use CD38 expression of remaining red blood cells (CD38 negative). Alternatively, use a fixed cut‐off point.
  The latter is possible if all instrument settings and staining protocols are standardized and results are reproducible. In our experimental setting, the chosen cut‐off is 10^2^ (see dashed line, Fig. 7B). Such a cut‐off had to be chosen because there is no robust control for CD38 negativity; such a cut‐off for negativity can only be chosen if instrument settings are standardized.
  Use FSC/SSC plot to gate the cluster of CD34+ blasts (exclude non‐specific cells with higher SSC and/or FSC, Fig. 7C); Figure 7D shows the population of CD34+CD38− stem cells (azure blue).

**Figure 8:**
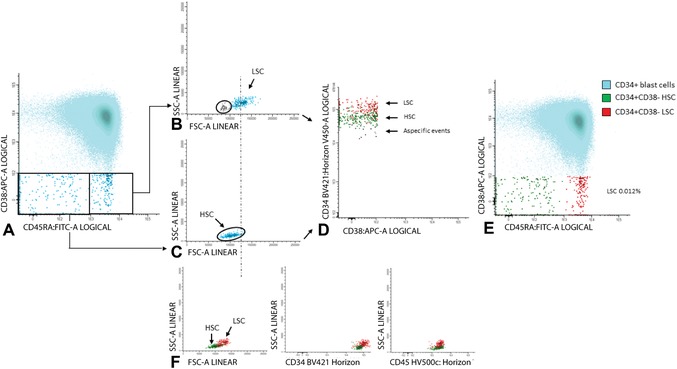
Gating of CD34+CD38− LSC cells using CD45RA as a stem cell marker. (**A**) This figure shows gating of the CD34+CD38− cells using CD45RA. (**B‐C**) Backgating of both the CD45RA positive and negative cell fractions in a FSC/SSC plot is shown in B and C, respectively. (**D**) Differences in CD34 expression between presumed LSC and HSC. (**E**) Final LSC results based on CD45RA as a stem cell marker. (**F**) Overview of the different secondary gating parameters is shown. A more detailed description of this figure can be found in Basic Protocol 2, step 7. In more detail: CD45RA negative and positive cell fractions were quite clearly separated (highlighted with a frame in A). Secondary gating parameters (FSC/SSC/CD34/CD45) were used to establish whether the presumed LSC and HSC populations were pure (this is further elucidated in the Critical Parameters and Troubleshooting section). B shows backgating of the CD45RA positive stem cells in a FSC/SSC plot. This shows that two populations with a different FSC and SSC can be discriminated within the CD45RA positive cell fraction. Backgating of the CD45RA positive FSC^low^ cells (marked in gray, B) in a CD34/CD38 plot shows that these cells have relatively low CD34 expression and are defined as a‐specific. C shows backgating of the CD45RA negative stem cells in an FSC/SSC plot. This backgating shows a pure CD45RA negative clustered FSC^low^ population, implying that there is little/no contamination with LSCs. D shows differences in CD34 expression between the LSC (in red), the presumed HSC (in green), and the earlier defined a‐specific events (in gray). Subsequently, the a‐specific events were removed from further analyses and E shows the final CD45RA expression results. F shows the results for all secondary gating used (FSC/SSC, CD34/SSC, and CD45/SSC), also showing that the CD45/SSC parameter does not contribute in this particular AML case. LSC, leukemic stem cell; HSC, hematopoietic stem cells; AML, acute myeloid leukemia.

**Figure 9:**
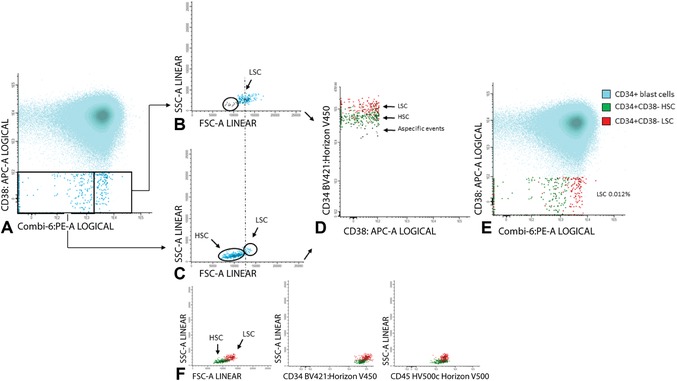
Gating of the CD34+CD38− LSC cells using the Combi‐6 marker. (**A**) This figure shows gating of the CD34+CD38**−** cells using the Combi‐6 marker. (**B‐C**) Backgating of both the Combi‐6 positive and negative cell fractions in a FSC/SSC plot is shown in B and C, respectively. (**D**) Differences in CD34 expression between presumed LSC and HSC. (**E**) Final results of Combi‐6 as a stem cell marker including FSC as a secondary gating parameter. (**F**) Overview of the different secondary gating parameters. A more detailed description of this figure can be found in Basic Protocol 2, step 7. In more detail: An example of LSC gating in the same AML using the Combi‐6 marker is shown in A. In contrast to CD45RA there is no clear separation between putative LSC and HSC. Possible Combi‐6 positive and Combi‐6 negative cells were globally defined in A (highlighted with a frame). B shows backgating of the Combi‐6 positive stem cells in a FSC/SSC plot. This shows that two populations with a different FSC and SSC can be discriminated within the Combi‐6 positive cell fraction. Similar to CD45RA, backgating of the Combi‐6 positive FSC^low^ cells (marked in gray, B) in a CD34/CD38 plot shows that these marker‐positive FSC^low^ cells have relatively low CD34 expression and are defined as a‐specific events. C shows backgating of the Combi‐6 negative stem cells in an FSC/SSC plot. In contrast to CD45RA, this backgating shows a Combi‐6 negative FSC^low^ population and a tiny Combi‐6 negative FSC^high^ population. Based on the FSC characteristics of the Combi‐6 positive stem cells (dotted line between B and C), there is a tiny fraction which should be defined as leukemic based on FSC as a secondary gating parameter (Terwijn et al., 2014). D shows the differences in CD34 expression between the LSC (in red), the presumed HSC (in green), and the earlier defined a‐specific events (in gray). Subsequently, the a‐specific events were removed from further analyses and E shows the final Combi‐6 expression results. F shows the results for all secondary gating used (FSC/SSC, CD34/SSC, and CD45/SSC), also showing that the CD45/SSC parameter does not contribute in this particular AML case. So, despite the initial poor separation between LSC and HSC (A), LSC and HSC can be fairly well distinguished using secondary gating parameters resulting in similar calculated LSC frequencies: 0.012% in both CD45RA and Combi‐6 analyses which is below the previously defined cut‐off of 0.03% (% of WBCs) for positivity at diagnosis (Zeijlemaker et al., 2019). Also, quite similar LSC/HSC ratios in the total CD34+CD38**−** compartment were found: 37:63 for CD45RA and 34:66 for Combi‐6. LSC, leukemic stem cell; HSC, hematopoietic stem cells.

7 Gate aberrancies on CD34+CD38− LSC cells: For this step, use the different stem cell markers as shown in Table 2 (CD45RA, Combi‐6, CD123, CD44, and CD33) to discriminate presumed normal hematopoietic stem cells (HSCs) from the LSCs.
  Examples of LSC gating are shown in Figures 8 and 9. Figure 8 shows an example where LSC and HSC can easily be defined using the CD45RA marker and Figure 9 shows a more difficult example using the Combi markers. A detailed description of the gating strategies in these cases is outlined in the legends of Figures 8 and 9.
  The background of the stem cell tube used (Table 2) is described in the Critical Parameters and Troubleshooting, Discrimination between LSCs and HSCs section. More detailed information, including examples about how to gate LSCs using these different stem cell markers, is found in Zeijlemaker et al. (2016a). Furthermore, more examples of how secondary parameters can be attributed to further define CD34+CD38− HSC and LSC are shown in Terwijn et al. (2014).
  Gating strategies to identify CD34+CD38− LSCs at follow‐up are identical compared to diagnosis, as described above in steps 5‐7.

## REAGENTS AND SOLUTIONS

### Lysing solution

Dissolve 1 part pharm‐lysing solution (BD Biosciences) in 10 parts Aqua Dest distilled water (e.g., Thermo Fisher Scientific). Store at room temperature for 1 day.
  As an alternative, a commercially available equivalent can be used.

### PBS

Dissolve PBS concentrate in demi water (e.g., Lenntech) in a ratio of 1:10. Per liter diluted PBS, add 0.5 g sodium azide (final concentration 0.05%). Mix 500 ml PBS/0.05% azide with 2.5 ml 20% (w/v) human serum albumin (HSA). Store at 4°C for up to 1 month.
  Final concentration HSA: 0.1%.

## COMMENTARY

### Background Information

In recent years, multiple studies have published on the prognostic value of MRD in AML (Hourigan, Gale, Gormley, Ossenkoppele, & Walter, 2017) and currently studies are ongoing in which therapy is adapted, guided by MRD results. Two different established modalities for detection of MRD are molecular techniques and multiparameter flow cytometry (MFC). Molecular‐based MRD is assessed by reverse transcriptase PCR (RT‐qPCR). Although different targets can be used for molecular MRD detection, the NPM1 mutation offers the most suitable target because it is very specific, sensitive, and stable (Ivey et al., 2016). However, only part of the AML cases is characterized by the NPM1 mutation and because MFC enables the analysis of MRD in the vast majority (>90%) of AML cases for the best prediction of an AML relapse, at present both NPM1‐MRD and MFC‐MRD are necessary (Zeijlemaker et al., 2019). Newer technologies, including next generation sequencing (NGS), are promising but still in a preliminary stage, although they have already been suggested to be potentially highly sensitive (Jongen‐Lavrencic et al., 2018; Thol et al., 2018). Although NGS has significant prognostic value, a recent publication showed that, at present, it cannot replace MFC‐MRD (Jongen‐Lavrencic et al., 2018). Therefore, for future residual disease assessment the combined use of NGS and MFC warrants further development. This protocol describes the method for the widely applicable MFC detection of both MRD and the small fraction of LSCs therein.

A possible alternative flow cytometric way to detect residual disease, which in its present form aims at quantification of the total leukemic load, is defining which part of the primitive (progenitor) population is made up by residual leukemic cells. The background of this is that cells characterized by the markers CD34 or CD117 or CD133 under normal non‐leukemia conditions are able to divide and to generate normal mature blood cells, while in AML part of such cells are able to propagate leukemia (Beghini et al., 2012; Blair & Sutherland, 2000; Blair, Hogge, & Sutherland, 1998; Quek et al., 2016). The LAIP‐approach can now be applied on the primitive blast cells, with MRD now defined as percentage aberrancy of the total progenitor compartment, instead of the total amount of WBCs as in the conventional MRD assay. In an earlier study, Terwijn and colleagues (2012) showed that this so‐called primitive marker MRD (PM‐MRD) harbors important prognostic impact, comparable to the conventional MRD assessment described in Basic Protocol 1 (Terwijn et al., 2013). If the prognostic value can be confirmed by other studies, the advantages are (1) hemodilution of bone marrow (BM) samples with peripheral blood offers a largely uncertain but disruptive factor in defining reliable MRD results. Although it is difficult to demonstrate hemodilution it can be an important cause of false‐negative MRD results. Hemodilution mainly affects the frequency of WBCs, and thereby MRD, but importantly it does not largely affect the composition of the primitive blast cells. Therefore using PM‐MRD largely annihilates the effects of hemodilution, thereby presumably reducing the number of false‐negative MRD results (Terwijn et al., 2012). (2) Moreover, PM‐MRD offers a method that can help in further standardization of the MRD assessment because omitting steps such as WBC gating in this approach automatically omits a source of variation. Because residual disease detection will play an increasing role in the treatment of AML, as well as in the establishment of efficacy of new therapies, such further standardization of the assay is necessary for future multicenter AML MRD studies.

Further improvement of predicting an AML relapse was accomplished by incorporation of the LSC frequency. There is growing evidence that a small fraction of leukemic cells with stem cell capacity are more therapy resistant compared to the majority of blast cells and it is suggested that outgrowth of these LSCs is ultimately responsible for the actual relapse (Becker & Jordan, 2011; Bonnet & Dick, 1997). Such LSCs may have different immunophenotypes (CD34+CD38+, CD34+CD38−, CD34−), however, CD34+CD38− LSCs have been shown to be most therapy resistant, both in vitro and in vivo, and leukemogenic (Bonnet & Dick, 1997; Costello et al., 2000; Ishikawa et al., 2007; van Rhenen et al., 2005). Moreover, in an earlier retrospective (Terwijn et al., 2014) and prospective study (Zeijlemaker et al., 2019), we showed the clinical relevance of these CD34+CD38− LSCs. Including the CD34+CD38− LSC frequency into the classical MRD frequency assessment strongly improves the prognostic impact of MRD frequency (Zeijlemaker et al., 2019). In future AML studies, therapeutic strategies may thus be based on the combined MRD and LSC results. One of the major problems with MRD and LSC assays is the occurrence of false negativity, i.e., part of patients who are MRD^negative^/LSC^negative^ may nevertheless relapse. Sensitivity of both assays can be further improved by further increasing the number of markers in the antibody panel and by further increasing the number WBCs analyzed. Therefore, with limited amounts of BM available, especially for the cell consuming LSC assay, reduction of the multiple‐tube approach to an 18‐color “one‐tube” approach (and including both an MRD and LSC panel) is warranted and currently being developed in our institute. It is expected that in the near future this will enable an even more efficient combined residual (stem cell) disease approach with increased sensitivity.

### Critical Parameters and Troubleshooting

#### Materials

For diagnostic LAIP and LSC determination, peripheral blood can be used because high percentages of blasts are often present in the peripheral blood at the time of diagnosis. However, for follow‐up MRD and LSC assessment, BM is preferred because the frequency of leukemic cells is usually lower in peripheral blood compared to BM (Maurillo et al., 2007; Zeijlemaker et al., 2016a). Nevertheless, usage of peripheral blood would be an attractive alternative source because BM acquisition is relatively invasive and time consuming. Three studies have shown that immunophenotypic MRD in peripheral blood of AML patients has prognostic value, similar to BM‐MRD (Guénot et al., 2019; Maurillo et al., 2007; Zeijlemaker et al., 2016a). Large multicenter studies and/or analyses are planned within ELN, which should confirm this and preferentially define MRD positivity in terms of cut‐off frequencies. It remains to be seen whether PM‐MRD in peripheral blood would also offer an option, because PM‐MRD seems largely independent of the source (BM or peripheral blood; Terwijn et al., 2012). However, these promising results need to be validated in future AML studies. For CD34+CD38− LSC detection, usage of peripheral blood is far from feasible yet, because it would require too many WBCs (likely >20 million) to detect the low frequent LSCs.

#### LAIP definition

Different LAIPs, including a different primitive marker (CD34, CD117, or CD133), are defined by different sensitivities and specificities. Total number of WBCs analyzed and the LAIP coverage of the leukemic blast cells (e.g., LAIP covers 80% of the blasts at diagnosis) are factors of importance for LAIP sensitivity. On the other hand, LAIP background expression, defined as expression of the LAIP on normal BM cells, is an important factor that influences LAIP specificity. High sensitivity and specificity values are of importance for reliability of the MRD assessment. Based on the above‐mentioned LAIP characteristics, the LAIPs with highest sensitivity and specificity are recommended for follow‐up MRD detection. However, the focus should not only be on the LAIPs with a high sensitivity and specificity at diagnosis because such LAIPs may disappear under therapy, while others persist or even emerge during follow‐up of the disease. The latter may not have been present at diagnosis or only at very low blast coverage (see also the Different from normal approach and immunophenotypic changes section). A more detailed overview of factors affecting the sensitivity and specificity of the MRD assay can be found in the recently published paper by Schuurhuis, Ossenkoppele, Kelder, and Cloos (2018).

#### Number of events

For MRD assessment the aim is to acquire 100,000 events for diagnosis samples and 1,000,000 events for follow‐up samples. The detection of CD34+CD38− LSCs within MRD is challenging because LSCs are often present in a very low frequency in follow‐up BM. Therefore, it is advisable to measure as many WBCs as possible to ensure a minimal reliability of LSC frequency. As shown in Table 2, only one tube is necessary for LSC detection both at diagnosis and follow‐up. This one‐tube approach with thirteen different antibodies theoretically enables the detection of a maximally possible number of WBCs. At present, LSC frequency assessment is sub‐optimal because of the necessity to use multiple tubes for MRD and LSC; in the setting presented in this paper five tubes are needed, four for MRD (Table 1) and one for LSC (Table 2). LSC and MRD will have to be combined to preferably one tube with at least 18 colors (see Background Information section) to offer the most optimal conditions for accurate assessment of both.

#### Cut‐off definition

Many studies have found that MFC‐MRD is an independent prognostic factor for outcome in AML (Buccisano et al., 2017). However, interestingly, many of these studies used different protocols for MFC‐MRD detection with often differences in lysing procedures, washing steps, and staining protocols. Moreover, different cut‐off levels were used to define MRD^low^ or MRD^negative^ patients with a relatively good prognosis and MRD^high^ or MRD^positive^ patients with a relatively poor prognosis. In all of these different studies, cut offs are used in a range of 0.035% to 0.15%; however, a generally accepted cut‐off value for MFC‐MRD is 0.1% (Schuurhuis et al., 2018).

In Basic Protocol 1 we outline details concerning our MFC‐MRD assessment used in the HOVON/SAKK studies whereby we also use the 0.1% cut‐off to define MRD positivity. Moreover, assessment of the LSC frequency gives important prognostic information both at diagnosis and at follow‐up. At diagnosis it is of importance to discriminate CD34^positive^ from CD34^negative^ AML patients. CD34^negative^ AML samples are characterized by the absence of leukemic CD34+ cells and thus no CD34+CD38− LSCs are present in these patients. These CD34^negative^ AML patients are characterized by the presence of only normal CD34+CD38− cells (HSCs) and a relatively good prognosis. More details concerning the definition and the prognostic value of CD34 status in AML is described elsewhere (Zeijlemaker et al., 2015). Within the CD34^positive^ AML patients, similar to MRD detection, a cut‐off is used to define LSC^high^ or LSC^positive^ patients with a relatively poor prognosis and LSC^low^ or LSC^negative^ patients with a relatively good prognosis. For diagnosis material a cut‐off of 0.03% (% of WBCs) was used to discriminate LSC^low^ from LSC^high^ samples. At present, follow‐up LSC assessment using one tube (Table 2) is sub‐optimal because part of the cells available at follow‐up have to be used for MRD frequency assessment in four tubes (Table 1). For the time being, we use a cut‐off of 0.0000%, implying that when one (or more) CD34+CD38− LSC(s) is/are present with 4 million WBCs analyzed, the patient is defined as LSC^positive^. LSC frequency turned out to have a prognostic impact (Zeijlemaker et al., 2019). Because such a cut‐off is highly questionable from a statistical point of view, we presently strive to develop a one‐tube approach (e.g., 18 color) for combined MRD and LSC assessment enabling much higher cell numbers for both the MRD and LSCs. The other approach is to further explore the prognostic possibilities of LSC frequencies at diagnosis (Zeijlemaker et al., 2019).

#### Discrimination between LSCs and HSCs

Because HSCs also reside within the CD45^dim^/CD34+CD38− cell compartment, for accurate LSC frequency assessment it is of utmost importance to be able to discriminate LSCs from HSCs, especially because both the frequency of LSCs and HSCs within this stem cell compartment may vary between 0% and 100% (Terwijn et al., 2014). At diagnosis and within the fraction of residual cells at follow‐up, aberrant cell surface marker expression can be used to discriminate LSCs from HSCs. Using such specific leukemia associated LSC immunophenotypes enables detection of LSCs in the vast majority of CD34^positive^ AML cases (Hanekamp, Cloos, & Schuurhuis, 2017). LSCs, as defined as aberrant marker positive CD34+CD38− cells, harbor specific molecular aberrancies (e.g., FLT3‐ITD, NPM1^mut^) and thus are indeed leukemic cells (Kersten et al., 2016; Schuurhuis et al., 2013; Terwijn et al., 2014).

Because, especially in follow‐up BM, numbers of CD34+CD38− cells can be (very) low, high sensitivity and specificity of the LSC assay is of importance. To further increase sensitivity and specificity, besides aberrant marker expression, secondary gating parameters (e.g., scatter properties, CD34 or CD45 expression; see also Figs. 8 and 9) can be used to discriminate LSCs from HSCs. This is based on the knowledge that LSCs sometimes have higher forward scatter (reflecting cell size) and sideward scatter (reflecting cell granularity) as compared to HSCs. Moreover, LSCs may differ from HSCs in CD34 or CD45 expression. Different examples of how these so‐called secondary gating parameters (FSC, SSC, CD34, and/or CD45) can attribute to further define the population of CD34+CD38− LSCs are shown in Figures 8 and 9 of this article and in Figures 1 and 3 of Terwijn et al. (2014).

Although these secondary parameters can help to define clusters of LSC cells, use of these secondary gating parameters is quite challenging, especially when low numbers of cells are available, and requires gating experience. Moreover, it is of importance to emphasize that there is large heterogeneity in aberrant marker expression on LSCs: Marker expression may differ between AML patients and even within an AML patient. Due to this large heterogeneity and to minimize the need to use the above mentioned secondary gating parameters, we designed an LSC tube that includes a broad panel of different markers (Table 2) that enables specific detection and accurate assessment of the frequency of CD34+CD38− LSCs in the majority of AML cases (Zeijlemaker et al., 2016b). In this panel the PE‐channel harbors six antibodies. The six antibodies all individually do not have the ability to stain HSCs (Zeijlemaker et al., 2016b). CD33, CD123, and CD44 show variable expression on HSCs and are in separate channels to reveal increased expression on LSCs. CD45RA has no expression on HSCs, but because it was added at a later time point to the panel, it was not included in the PE channel. CD34, CD45, CD38 are the core antigens for CD34+CD38− stem cell identification.

#### Different from normal approach and immunophenotypic changes

For proper MRD detection, four tubes with antibodies are measured both at time of diagnosis and follow‐up (Table 1). This enables MRD detection via both the LAIP approach (as outlined in this protocol) and the different from normal approach (DfN). The LAIP approach encompasses the detection of LAIPs in follow‐up BM that were present at the time of diagnosis. However, an important limitation of this approach is that when the focus is only on present LAIPs at diagnosis, upcoming AML populations can be missed; this may (at least partially) explain why some MRD^negative^ patients will still experience a relapse. Although the clinical relevance for prognostication is unknown for these upcoming AML populations, it is advisable to measure all four MRD tubes at follow‐up if enough BM is available. In the DfN approach, aberrant differentiation patterns are identified and from these aberrant patterns different LAIPs can be extracted allowing subsequent quantification. It should be stressed at this point that the ELN MRD Working Party emphasizes integration of the LAIP and DfN approaches (Schuurhuis et al., 2018). Importantly, the DfN approach can be used when diagnosis material is missing or in the few cases where no LAIPs are present at diagnosis.

With this four‐tubes approach, all immunophenotypic markers are measured at follow‐up and this enables detection of AML cells that have undergone an immunophenotypic shift, including emerging new populations. Such immunophenotypic changes, defined as changes in the expression of cell surface markers between diagnosis and relapse, are reported in several studies and reviewed by Zeijlemaker, Gratama, and Schuurhuis (2014). Detection of CD34+CD38− LSCs at follow‐up is challenging due to the often very low frequency of these cells during follow‐up. However, with the one‐tube approach as shown in Table 2, similar to MRD, all markers (or combinations) are measured both at diagnosis and at follow‐up. Therefore, this enables detection of immunophenotypic changes between diagnosis and relapse, including upcoming CD34+CD38− leukemic populations.

### Understanding Results

Although different cut‐off levels can be used to define MRD^low^ or MRD^negative^ patients and MRD^high^ or MRD^positive^ patients, we use the generally accepted MRD cut‐off of 0.1% (Schuurhuis et al., 2018). This implies that patients above the cut‐off have a relatively poor prognosis and patients below this cut‐off have a relatively good prognosis.

For the CD34+CD38− LSC assessment at diagnosis, a cut‐off of 0.03% (% of WBCs) was used to discriminate LSC^low^ from LSC^high^ samples, whereby LSC^high^ patients have a relatively poor prognosis as compared to LSC^low^ patients. For the LSC assessment at follow‐up, a cut‐off of 0.0000% is used. More information concerning the use of these cut‐offs is outlined in the paragraph above (Critical Parameters and Troubleshooting, Cut‐off definition section).

### Time Considerations

In Basic Protocol 1, processing of a single sample for MRD assessment takes roughly 150 min. Basic Protocol 2, which describes detection of CD34+CD38− cells, also takes roughly 180 min for processing one sample.
